# Human Induced Pluripotent Stem Cells in Rare Neurological Disorders: Opportunities and Challenges for Disease Modeling and Drug Discovery

**DOI:** 10.1155/sci/1130043

**Published:** 2026-10-21

**Authors:** Anja Harej Hrkać, Valentino Rački, Andrej Belančić, Jasna Kriz, Dinko Vitezić, Jasenka Mršić-Pelčić, Ganesh Acharya, Mukesh Varshney, Kristina Pilipović

**Affiliations:** ^1^ Department of Basic and Clinical Pharmacology and Toxicology Faculty of Medicine University of Rijeka Rijeka Croatia uniri.hr; ^2^ Clinic of Neurology Clinical Hospital Center Rijeka Rijeka Croatia kbc-rijeka.hr; ^3^ Research Center of the Quebec University Institute of Mental Health (CRIUSMQ) Laval University Quebec City Canada ulaval.ca; ^4^ Center for Embryonic and Prenatal Precision Medicine (PRECISE) Division of Obstetrics and Gynecology Department of Clinical Science, Intervention and Technology Karolinska Institutet Stockholm Sweden ki.se; ^5^ Center for Fetal Medicine Karolinska University Hospital Stockholm Sweden karolinska.se; ^6^ Division of Clinical Microbiology Department of Laboratory Medicine Karolinska Institutet Stockholm Sweden ki.se

**Keywords:** disease models, genetic heterogeneity, induced pluripotent stem cells, nervous system diseases, rare diseases

## Abstract

Rare disease is a condition that affects a small fraction of the population. In most cases, it has a known genetic cause but could also be associated with other contributing factors. As the affected individuals may exhibit diverse symptoms due to the genetic and phenotypic heterogeneity, non‐ or misdiagnosis of rare diseases is common. Their low incidence and prevalence present an obstacle to developing new therapies despite a major unmet need with only a few treatment options available. This is particularly relevant to rare neurological disorders as there are major difficulties in studying the mechanisms of initiation and progression of the central nervous system (CNS) disorders. Recent advances in biotechnology, specifically the use of human induced pluripotent stem cells (iPSCs) for modeling diseases, have been instrumental in providing a flexible platform that not only reflects the effects of genetic disturbances on the development of CNS disorders but also is capable of providing insights into complex multifactorial disorders. Whether the human iPSCs are harvested from the affected individuals or using the gene‐editing tools on cells from healthy individuals, valuable models can now be made available for studying these orphan conditions. In this narrative review, we explore the benefits of using human iPSC models over the existing platforms of other primary cell lines and animal models for the disease modeling and drug discovery and discuss the limitations and future aspects of the iPSC use in the investigation of rare neurological disorders.

## 1. Introduction

Rare diseases are defined in the European Union and by the World Health Organization as conditions affecting fewer than 1 in 2000 people [1]. While individually uncommon, they collectively affect ~27–36 million people in Europe and around 300 million worldwide [1, 2]. More than 7000 rare diseases have been identified to date, with ~80% having a genetic origin [1], and many manifest early in life with significant morbidity and mortality. Despite their individual rarity, these diseases often share common challenges: genetic heterogeneity, complex clinical manifestations, delayed diagnoses, and limited therapeutic options. Historically, treatment was limited to symptomatic relief or palliative care, as therapeutic development for rare diseases faced substantial obstacles such as small patient populations, high research costs, and limited commercial incentives [2, 3]. To address these gaps, the EU implemented the Orphan Drug Regulation (EC Number 141/2000) to foster research and development by providing regulatory and financial incentives. Consequently, in the period between 2000 and 2024, 261 orphan medicines for rare diseases were approved for marketing, and the Committee for Orphan Medicinal Products (EMA COMP) granted orphan designation to 3012 products, illustrating a dynamic research environment and growing therapeutic possibilities [4–6].

A significant breakthrough in the understanding and treatment of rare neurological diseases (RNDs) has emerged from the discovery and application of cellular reprogramming to create human induced pluripotent stem cell (iPSC) models [7, 8]. iPSCs are a subtype of stem cells that were originally developed by Prof. Shinya, for which he was awarded the Nobel Prize in Physiology or Medicine in 2012 [9, 10]. Specifically, iPSCs differ from other pluripotent cells, such as the embryonic stem cells, in that they are generated from adult somatic cells (commonly used are skin or blood cells) by introducing reprogramming factors, making it possible to first reprogram them back into a pluripotent state, followed by differentiation into various cells of the body such as neurons, cardiomyocytes, cartilage, blood cells, or hepatocytes. These iPSCs can also be used for the creation of organoids, self‐organized, three‐dimensional (3D) structures that can mimic the structure and physiology of human organs [10–13]. Patient‐derived models faithfully replicate disease‐specific mutations and cellular environments, providing invaluable platforms for developmental and functional analyses. iPSC technology enables researchers to study disease pathogenesis in human neuronal networks, screen for therapeutic agents, and explore novel molecular targets [7]. The discovery and application of cellular reprogramming to create novel iPSC models have revolutionized the rare disease community, bridging the gap between basic research and clinical application. These advances affirm that modern medicine now holds more resources to address conditions once deemed incurable.

Although iPSC approaches are relevant across rare diseases, this review focuses on rare neurological disorders because disease‐relevant neural tissue is largely inaccessible during life, clinical phenotypes often depend on interactions among neurons and glia, and substantial genetic and phenotypic heterogeneity complicates typical model systems. Patient‐specific iPSC‐derived neural cultures, organoids, and isogenic controls can support the investigation of disease mechanisms and therapeutic responses in a human cellular context. Accordingly, broader rare‐disease concepts are used only as background, whereas the following sections focus on rare neurological disorders [11–15].

## 2. RNDs—Unresolved Issue in Modern Medicine

RNDs constitute a significant and often underrecognized challenge within modern medicine. Most rare diseases have prominent neurological symptoms, which have a drastic effect on survivability, disability, and quality of life [16]. RNDs encompass a wide array of conditions such as leukodystrophies, mitochondrial encephalopathies, inherited ataxias, spinal muscular atrophy (SMA), Rett syndrome, and numerous other monogenic and complex disorders [17]. These conditions are often present from early childhood, follow a progressive and disabling trajectory, and result in profound neurological impairment. The human, social, and economic impacts of RNDs are substantial, extending far beyond the affected individual to their caregivers, families, and the healthcare system at large [18].

Despite remarkable advances in genomics, molecular diagnostics, and neuroimaging, RNDs remain among the most underserved entities in modern clinical practice. One of the most pressing challenges is the so‐called “diagnostic odyssey,” referring to the extended period that many patients endure before receiving a definitive diagnosis [19]. This delay in diagnosis is often measured in years and can be attributed to several factors, such as phenotypic overlap with more common disorders, limited clinical awareness of rare conditions, and restricted access to comprehensive genetic testing [20]. Still, the diagnosis of these disorders has improved significantly with the advances in genetic sequencing [19]. However, even when a diagnosis is eventually established, it often comes too late to alter the course of disease meaningfully, especially in disorders with early‐onset neurodegeneration [21]. The emerging role of noninvasive early prenatal diagnosis by whole genome sequencing of cell‐free fetal DNA (cffDNA) from maternal blood cannot be overemphasized in this regard [22].

While the successful development and implementation of gene‐based therapies for SMA, such as nusinersen, risdiplam, and onasemnogene abeparvovec, provide a compelling proof of concept, these interventions remain the exception rather than the rule [21]. Most of these disorders currently lack any disease‐modifying therapy and are still managed symptomatically, with treatment strategies that are largely palliative and supportive rather than curative or preventive while depending on well‐developed multidisciplinary teams [23]. Pharmaceutical investment in rare disease drug development is further hindered by small patient populations, heterogeneous clinical manifestations, and a paucity of validated biomarkers, all of which complicate the design and execution of adequately powered clinical trials [24]. As a result, many promising therapeutic avenues remain unexplored, and the transition from preclinical discovery to clinical application remains exceedingly slow.

### 2.1. Limitations of Current Research Tools

The research infrastructure and tools currently available to study RNDs are limited [25]. Access to relevant human neural tissue is inherently constrained by the inaccessibility of the brain and spinal cord during life. Postmortem tissue, while valuable, often represents advanced disease stages and provides limited insight into early pathophysiological events [26]. In the absence of human neural material, researchers have traditionally relied on animal models [27]. Even though these models contribute to a basic understanding of disease mechanisms, they often fail to capture the full spectrum of human neurodevelopmental and neurodegenerative processes. For example, there is limited replication of precise genetic and epigenetic contexts in human diseases, as well as the complexity of neuronal‐glial interactions, which lead to poor translational efficacy of therapeutics that appear promising in preclinical studies [28]. Furthermore, even well‐characterized monogenic diseases often display substantial interindividual phenotypic variability, the origins of which remain poorly understood. This variability may arise from the influence of genetic modifiers, environmental exposures, or unknown biological processes that are difficult to model in current systems [29].

Although next‐generation sequencing technologies have significantly expanded our capacity to identify disease‐causing mutations, the translation of genetic discoveries into mechanistic insights and actionable therapeutic targets remains slow. A major factor in this is the absence of scalable, physiologically relevant, and patient‐specific platforms capable of recapitulating human neural function in a controlled in vitro setting [14]. This translational gap has prompted a shift in disease modeling, particularly with the advent of pluripotent stem cell reprogramming, first described by Takahashi and Yamanaka in 2006 [9]. This technology enables the creation of patient‐specific neural cultures that preserve the individual’s genetic background, including both causative mutations and modifying factors. As such, iPSCs have become a tool for modeling RNDs, particularly those with heterogeneous presentations or subtle cell‐type–specific dysfunction [9, 14].

### 2.2. Unresolved Issues Through Diseases

Several well‐characterized RNDs provide good examples of the unresolved clinical and research challenges in this field. Notably, SMA, caused by biallelic mutations in the *SMN1* gene, represents a success story in rare disease therapeutics. The development of antisense oligonucleotides and gene replacement therapies has significantly altered the disease trajectory for many patients [30]. Nevertheless, the cost and limited global accessibility of these treatments raise critical ethical and practical questions regarding health equity, particularly in lower‐resource settings [31, 32]. Niemann–Pick disease type C, a lysosomal storage disorder with highly variable neuropsychiatric and motor manifestations, is frequently misdiagnosed or diagnosed late, with the average diagnostic delay exceeding 5 years [33]. Novel treatments emerged recently that hold great promise but will require long‐term follow‐up and, as with SMA, hold an impressive cost that will limit their adoption worldwide [34].

Polymerase (DNA) Gamma (POLG)‐related mitochondrial disorders, resulting from mutations in the gene encoding mitochondrial DNA polymerase gamma, offer another compelling example. These disorders encompass a broad phenotypic spectrum, including childhood‐onset encephalopathies, myocerebellar syndromes, and progressive external ophthalmoplegia. The phenotypic variability is often seen within families, reflecting complex gene–environment and epigenetic interactions. Current animal models fail to capture this heterogeneity, and efforts to translate molecular insights into targeted treatments have been largely unsuccessful [35]. A small open‐label, single‐arm phase 2 trial was recently published focusing on deoxycytidine and deoxythymidine, which is promising but also highlights the challenges of having well‐defined clinical data due to the forced study design through disease rarity [36].

Rett syndrome, a prototypical monogenic neurodevelopmental disorder caused by mutations in the *MECP2* gene, illustrates the difficulty of therapeutic translation despite a clear genetic etiology. The disorder’s pathogenesis involves both neuronal and glial dysfunction, highlighting the need for models capable of recapitulating the full cellular complexity of the human central nervous system (CNS) [37]. Trofinetide, a synthetic analog of the amino‐terminal tripeptide of insulin‐like growth factor 1, was approved by the FDA for the treatment of Rett syndrome and, in a randomized phase 3 trial, demonstrated statistically significant improvements in Rett syndrome–specific clinical measures compared with placebo [38].

In summary, RNDs remain an unresolved issue in modern medicine, marked by diagnostic delays, limited therapeutic options, and substantial gaps in translational research. Despite advances in genetic diagnostics and isolated therapeutic successes, such as those in SMA and Rett syndrome, the majority of RNDs continue to lack disease‐modifying treatments. Central to this are limitations in currently available research tools, including the inaccessibility of human neural tissue and the insufficient translational fidelity of animal models. The emergence of iPSC technology offers a promising response to these challenges by enabling the generation of patient‐specific, physiologically relevant models that can bridge the gap between molecular discovery and clinical application.

## 3. The Use of Human iPSC Cells for Modeling RNDs: Available Platforms, Benefits, andLimitations

Human iPSC technology has transformed the modeling of rare neurological disorders and strengthened disease‐focused drug discovery [9]. The ability to reprogram adult somatic cells into a pluripotent state using defined transcription factors has opened unprecedented opportunities for studying human‐specific disease mechanisms that were previously inaccessible due to limitations of animal models and ethical constraints surrounding embryonic stem cell research [9]. In the context of RNDs, where patient populations are small and disease mechanisms are often poorly understood, the iPSC technology offers a unique platform for generating patient‐specific cellular models that can recapitulate disease pathology in vitro (Figure 1) [11].

**Figure 1:**
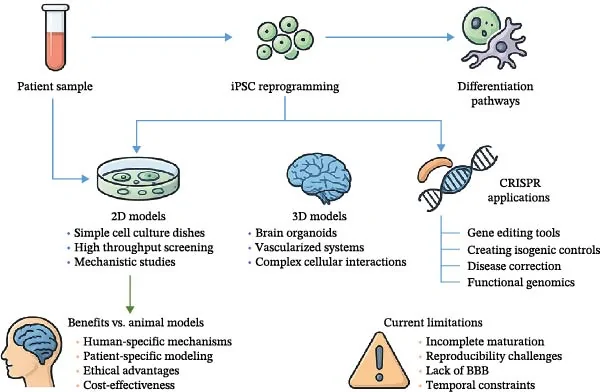
iPSC disease modeling in rare neurological disorders. The workflow and key components of using iPSCs for modeling rare neurological diseases. Starting from a patient‐derived sample, cells are reprogrammed into iPSCs and then directed into specific neural lineages via defined differentiation pathways. iPSCs can be used to generate both 2D models—such as simple cell culture systems for mechanistic studies and high‐throughput drug screening—and more complex 3D models, including brain organoids, vascularized systems, and complex cellular interactions. CRISPR‐based gene editing applications are also integrated allowing for the creation of isogenic controls, correction of disease‐causing mutations, and functional genomics investigations. The major benefits of iPSC‐based models over traditional animal models include species‐specific relevance, patient‐specificity, ethical advantages, and cost‐effectiveness. Despite these advantages, several limitations persist, such as incomplete cellular maturation, limited blood‐brain barrier (BBB) modeling, reproducibility issues, and constraints related to modeling long‐term disease progression.

The CNS presents particular challenges for disease modeling due to its complexity, the postmitotic nature of neurons, and intricate interactions between multiple cell types, including neurons, astrocytes, microglia, and oligodendrocytes [12]. Traditional approaches to studying neurological diseases have relied heavily on animal models, which often fail to fully recapitulate human‐specific disease mechanisms and drug responses [39]. The emergence of iPSC technology has provided researchers with tools to generate human neural cells from patients with rare neurological disorders, enabling the study of disease initiation, progression, and potential therapeutic interventions in a human cellular context [13].

This section examines the current state of iPSC‐based disease modeling platforms, with particular emphasis on their applications in rare neurological disorders (Figure 1). We explore the evolution from two‐dimensional (2D) culture systems to sophisticated 3D organoid models, analyze the advantages of iPSC models over traditional animal and primary cell line approaches, acknowledge current limitations that must be addressed for successful clinical translation, and discuss the transformative potential of CRISPR/genome editing technologies in enhancing disease modeling capabilities.

### 3.1. Platforms for Drug Discovery Using Human iPSC Disease Modeling

The development of robust and reproducible platforms for iPSC‐based disease modeling has emerged as a critical foundation for advancing drug discovery for rare neurological disorders [14]. Contemporary iPSC disease modeling platforms have evolved significantly beyond simple monolayer cultures to encompass sophisticated multicellular systems that better reflect the complexity of the human brain [40]. The development of standardized protocols for iPSC generation has been crucial, with our laboratory demonstrating the generation of nonviral integration‐free human iPSC lines under defined xeno‐free and feeder‐free conditions, providing a foundation for reproducible disease modeling [41].

One of the most significant advances in platform development has been the creation of long‐term culture systems that can maintain cellular viability and functionality over extended periods [42]. These platforms enable researchers to study complex cellular interactions fundamental to neurological disease pathogenesis, incorporating microglia, astrocytes, and vascular components into organoid systems [43]. The integration of multiple cell types within single platform systems has enabled more physiologically relevant environments for studying neuroinflammation, synaptic dysfunction, and neurodegenerative processes that characterize many rare neurological disorders [44].

Platform standardization and reproducibility have become increasingly important as the field moves toward clinical applications [15]. The establishment of core facilities and standardized protocols for iPSC generation, maintenance, and differentiation has been essential for ensuring consistent results across different laboratories and research groups [45]. These standardized approaches are particularly critical for rare disease research, where patient samples may be limited, and interlaboratory collaboration is essential for advancing understanding of disease mechanisms [46].

The scalability of iPSC platforms for high‐throughput screening applications represents another key consideration for drug discovery efforts [47]. Modern platforms must balance the complexity required to model disease pathology with the throughput necessary for screening large compound libraries or testing multiple experimental conditions [48]. Recent advances in automation and miniaturization have enabled the development of platforms that can maintain biological relevance while supporting the screening requirements of pharmaceutical development programs [49].

Quality control measures have become integral to platform development, with emphasis on maintaining genetic stability and pluripotency markers throughout culture expansion [50]. The implementation of comprehensive characterization protocols ensures that iPSC lines retain their differentiation potential and genetic integrity, which is essential for reliable disease modeling [51]. Future directions in platform development are likely to focus on increasing physiological relevance through the incorporation of additional cellular components while maintaining the reproducibility and scalability required for drug discovery applications [52].

### 3.2. Generating 2D Neural Cells and 3D Organoids From iPSCs

Reproducible disease modeling begins with the iPSC line itself. Patient somatic cells, most commonly dermal fibroblasts or peripheral blood mononuclear cells, are reprogrammed by transient delivery of the Yamanaka factors using nonintegrating systems such as Sendai virus, episomal plasmids, or synthetic mRNA. Lines intended for cross‐line comparison should be derived and maintained under defined, xeno‐free, and feeder‐free conditions to limit batch variability; we have previously described the derivation of such integration‐free lines from human fibroblasts [41]. Before differentiation, lines are verified for pluripotency marker expression, trilineage differentiation potential, donor identity by short tandem repeat profiling, and genomic integrity by karyotyping or SNP array, and passage number is recorded, since culture‐acquired variants accumulate with extended expansion.

2D neural differentiation follows one of two strategies. Directed differentiation recapitulates developmental patterning: Dual inhibition of SMAD signaling converts iPSCs into neuroepithelium within approximately 2 weeks [53], after which regional identity is imposed by morphogens. Withholding caudalizing and ventralizing cues yields dorsal forebrain progenitors and subsequently cortical glutamatergic neurons [54]; sonic hedgehog (SHH) agonism combined with WNT inhibition generates medial ganglionic eminence‐like progenitors and forebrain GABAergic interneurons, which require prolonged culture to mature [55]; retinoic acid with SHH agonism specifies spinal motor neurons [56]; and floor‐plate induction using SHH, FGF8, and WNT activation produces midbrain dopaminergic neurons [57], a protocol we have applied to examine pharmacological effects on dopaminergic specification [58]. The second strategy bypasses patterning altogether: Inducible expression of NGN2 converts iPSCs into a comparatively homogeneous population of excitatory neurons within 2 weeks [59], and transcription factor‐based protocols are now standardized for routine use [60]. Glial populations are obtained over longer timescales, astrocytes from expanded neural progenitors [61] and microglia through a hematopoietic progenitor intermediate [62], and can be recombined with neurons in defined cocultures. Protocol choice at this stage determines what can be asked later: Directed differentiation preserves the developmental sequence and produces progenitors that remain expandable, whereas transcription factor induction sacrifices that intermediate for speed and homogeneity, so the two are not interchangeable when progenitor‐stage phenotypes are part of the question.

3D protocols differ chiefly in how much patterning is imposed. Unguided cerebral organoids rely on intrinsic self‐organization: Embryoid bodies are induced towards neuroectoderm, embedded in the extracellular matrix to support neuroepithelial expansion, and maintained under agitation, generating tissues that contain multiple regional identities [42, 63]. Guided protocols substitute defined morphogen exposure for self‐organization and produce dorsal forebrain, ventral forebrain, midbrain, or spinal organoids of more consistent composition [64, 65], and region‐specific organoids can subsequently be fused into assembloids to reconstruct interneuron migration and inter‐regional connectivity [65]. Extending viability and complexity requires further intervention: Sectioning organoids and culturing them at an air–liquid interface relieves the necrotic core and supports axon tract formation with functional output [66], while codevelopment with endothelial cells or induced ETV2 expression generates vascular‐like networks [67]. Organoid coculture is also used to introduce exogenous cell populations, as in our work seeding patient‐derived glioblastoma cells onto iPSC‐derived brain organoids [68]. Variability between batches remains the principal constraint, although directed protocols reproducibly generate comparable cellular composition across lines and differentiations [69]. Matrix and medium composition are not incidental to these protocols: Matrix embedding governs neuroepithelial expansion and the reproducibility of early morphology, while the switch from neural induction to maturation medium sets the timing of neurogenesis, and both should be reported in sufficient detail for a protocol to be repeated.

Protocol choice should follow the intended readout. Monolayer cultures of a defined, near‐homogeneous cell type are appropriate where the phenotype is cell‐autonomous and the experiment requires replicate number, throughput, or precise pharmacological control, which applies to most of the motor neuron, GABAergic neuron, and Schwann cell phenotypes discussed in Section 4. Organoids are warranted where the phenotype depends on tissue architecture, progenitor dynamics, or interaction between cell classes, as in the progenitor and migration defects of Miller–Dieker syndrome or the neuron–glia interactions reported in the tuberous sclerosis complex. Timeline is a practical constraint: Transcription factor induction delivers neurons within weeks, directed cortical differentiation within 2 to 3 months, and organoid phenotypes that depend on maturation may require 6 months or longer. Where the two formats address the same disorder, they are best treated as complementary rather than alternative, with the monolayer culture establishing the cell‐autonomous component of a phenotype and the organoid testing whether it persists in a tissue context. Table 1 summarizes the principal protocols, their approximate duration, and their main applications. Key 2D and 3D culture protocols for neural cells and organoid derivation are described, with culture duration and applications, in Table S1.

**Table 1:** Summary of the use of induced pluripotent stem cells (iPSCs) for modeling of selected rare neurological diseases.

Disease	Disease mechanism	Patient iPSC source	Differentiated cell type(s)	Main applications
Spinal muscular atrophy	SMN deficiency with motor‐neuron degeneration	Fibroblasts	Motor neurons	Mechanism [70, 71], drug screening [72]
Hereditary spastic paraplegia	Corticospinal axon degeneration	Fibroblasts	Motor neurons	Pathology [73], treatment testing [74]
Friedreich’s ataxia	Frataxin deficiency and mitochondrial dysfunction	Fibroblasts	Neurons	Pathogenesis [75], drug testing [76–79]
Pompe disease	GAA deficiency with lysosomal glycogen accumulation	Fibroblasts	Myocytes, cardiomyocytes	Phenotyping [80–82], disease modeling [83]
Dravet syndrome	*SCN1A* haploinsufficiency with interneuron dysfunction and neuronal hyperexcitability	Skin/blood cells	Neurons, cardiomyocytes	Multisystem study [84]
Charcot‐Marie‐Tooth disease	Peripheral nerve demyelination and/or axonal loss	Fibroblasts, lymphoblastoid cell lines, T lymphocytes	Neurons and glial cells, including neural crest cells, Schwann cells	Gene therapy [85], disease modeling [86–88]
Tuberous sclerosis complex	Loss of *TSC1* or *TSC2* with mTOR hyperactivation and hamartoma formation	Fibroblasts, PBMCs, or blood‐derived cells carrying heterozygous or mosaic *TSC1/TSC2* mutations	NPCs, NCCs, Purkinje‐cell–like neurons, astrocytes, organoids/cortical spheroids	Pathology, drug target screening [89]
Rett syndrome	*MECP2*‐related dysregulation of neuronal development	Fibroblasts	Neurons, NPCs, astrocytes	Mechanistic studies, drug screening [90–98]
Sturge–Weber, others	Mosaic vascular malformations due to *GNAQ* activation	–	–	In development, less reported

Abbreviations: GAA, acid alpha‐glucosidase; GNAQ, G protein subunit alpha q; MECP2, methyl‐CpG binding protein 2; mTOR, mechanistic target of rapamycin; NCCs, neural crest cells; NPCs, neural progenitor cells; PBMCs, peripheral blood mononuclear cells; SCN1A, sodium voltage‐gated channel alpha subunit 1; SMN, survival of motor neuron; TSC1/2, tuberous sclerosis complex 1/2.

### 3.3. Application of 2D and 3D iPSC Models for RND Modeling and Drug Discovery

2D iPSC–derived neural cultures provide tractable systems for examining cell‐type–specific phenotypes, including altered differentiation, synaptic function, neuronal excitability, and cellular stress responses in rare neurological disorders. Their relative simplicity supports reproducible mechanistic studies and scalable compound screening, particularly when patient‐derived lines and isogenic controls are used. In this review, 2D iPSC–derived models are discussed in relation to SMA, Rett syndrome, Dravet syndrome, Friedreich’s ataxia, Charcot‐Marie‐Tooth disease (CMT), and tuberous sclerosis complex [70, 71, 75–78, 80–82, 85–126].

The advantages of 2D systems extend beyond their technical simplicity to include their compatibility with automated screening platforms and their ability to generate large datasets for statistical analysis [127]. These characteristics make 2D models particularly valuable for pharmaceutical companies conducting large‐scale drug discovery programs, where the ability to test thousands of compounds efficiently is essential for identifying promising therapeutic candidates [128]. However, the limitations of 2D models have become increasingly apparent as our understanding of neurological disease complexity has advanced [129].

Within rare neurological disorders, 2D iPSC–derived neural cultures are particularly useful when phenotypes can be assigned to a defined cell type. For example, patient‐derived motor neurons have revealed selective vulnerability in SMA, whereas neuronal cultures from Rett and Dravet syndrome permit evaluation of mutation‐associated abnormalities in neuronal differentiation, synaptic function, and excitability. Such assays can be combined with isogenic controls and quantitative readouts to support mechanistic studies and early‐stage drug screening [70, 71, 90–103, 130].

In rare neurological disorders, these systems complement 2D cultures by enabling the study of disease‐relevant cellular interactions and developmental processes; examples discussed in this review include Miller–Dieker syndrome, Dravet syndrome, Friedreich’s ataxia, and tuberous sclerosis complex [76, 89, 113–126, 130, 131]. Their use should be balanced against recognized limitations in maturation, reproducibility, and throughput.

The development of brain organoids has been particularly transformative for studying neurodevelopmental disorders and neurodegenerative diseases, where tissue architecture and cellular interactions play crucial roles in disease pathogenesis [132]. These advanced 3D models have successfully recapitulated multiple disease pathologies, including amyloid plaque–like aggregates, tau tangle–like aggregates, neuroinflammation, and synaptic dysfunction [133]. The validation of organoid systems using FDA‐approved therapeutics has demonstrated the potential of these models to predict therapeutic efficacy and identify novel drug targets [134].

The integration of advanced imaging and analysis techniques with 3D models has further enhanced their utility for drug discovery [135]. High‐content imaging platforms combined with automated analysis algorithms enable researchers to quantify complex phenotypes and drug responses in 3D systems, bridging the gap between biological relevance and screening throughput [131]. Manufacturing considerations have become increasingly important as 3D models move toward clinical applications, with emphasis on developing uniform organoids with demonstrated homogeneity in both morphology and gene expression [69]. Future developments are likely to focus on combining the advantages of both approaches through integrated experimental strategies that maximize the strengths of each modeling platform [136].

### 3.4. Benefits of Using iPSC Models Over Animal and Primary Cell Line Models

The adoption of iPSC‐based disease models represents a paradigm shift in neurological disease research, offering substantial advantages over traditional animal models and primary cell line approaches [137]. One of the most significant advantages of iPSC models is their ability to capture human‐specific disease mechanisms and cellular responses that may not be adequately represented in animal models [138]. The fundamental species differences between humans and commonly used laboratory animals, particularly in brain development, neuronal connectivity, and immune responses, have contributed to the high failure rate of neurological therapeutics that show promise in animal studies but fail in human clinical trials [139].

The main advantage of iPSCs lies in their capacity to allow researchers to study specialized cell types that can be directly generated from individual patients, thus providing unprecedented access to disease‐relevant cellular models [140]. This patient‐specific approach is particularly valuable for RNDs, where the genetic heterogeneity of patient populations makes it essential to study disease mechanisms across multiple genetic backgrounds [141]. Unlike animal models, which typically represent single genetic variants or artificially introduced mutations, iPSC models can capture the full spectrum of genetic diversity present in human patient populations [142].

The ability to generate patient‐specific models has profound implications for understanding disease heterogeneity and developing personalized therapeutic approaches [143]. RNDs often exhibit significant phenotypic variability even among patients carrying the same genetic mutation, reflecting the influence of genetic modifiers, environmental factors, and epigenetic regulation [144]. iPSC models enable researchers to study these patient‐specific differences in a controlled experimental setting, providing insights into factors that influence disease severity and therapeutic response [145].

iPSC models also offer significant advantages in terms of accessibility and scalability compared to primary cell line approaches [53]. Primary neural cells, particularly human neurons and glial cells, are extremely difficult to obtain and maintain in culture, limiting their utility for large‐scale studies [146]. The postmitotic nature of neurons means that primary cultures have limited proliferative capacity and short lifespans, making them unsuitable for extended studies or high‐throughput screening applications [147]. In contrast, iPSCs can be expanded indefinitely while maintaining their pluripotent state, providing a virtually unlimited source of cells for differentiation into disease‐relevant cell types [148].

The reproducibility advantages of iPSC models over animal studies are particularly important for rare disease research, where patient populations are small and research resources are limited [149]. Animal studies often suffer from significant variability due to genetic background differences, environmental factors, and experimental conditions that can be difficult to control across different laboratories [150]. iPSC models, when properly standardized, can provide more consistent and reproducible results, enabling more reliable comparisons between studies and facilitating collaborative research efforts [151].

Ethical considerations also favor the use of iPSC models over animal approaches, particularly as public awareness of animal welfare issues continues to grow and regulatory agencies increasingly emphasize the principles of replacement, reduction, and refinement in animal research [152]. iPSC models offer a complete replacement for animal models in many experimental contexts, eliminating animal welfare concerns while potentially providing more relevant human data [153]. The cost‐effectiveness of iPSC models compared to animal studies represents another significant advantage, particularly for academic research groups and smaller biotechnology companies working on rare diseases [154]. The temporal advantages of iPSC models are particularly relevant for drug discovery applications, where the ability to rapidly test therapeutic hypotheses can significantly accelerate the development timeline [155].

### 3.5. Limitations of iPSC‐Based Models for CNS Drug Discovery

Despite the significant advantages offered by iPSC‐based models for studying rare neurological disorders, several important limitations must be acknowledged and addressed to realize their full potential for CNS drug discovery [156]. One of the most fundamental limitations of current iPSC models is their inability to fully recapitulate the complexity of the human brain [157]. While significant advances have been made in developing multicellular organoid systems, these models still fall short of reproducing the intricate multidimensional architecture, cellular diversity, and functional connectivity that characterize the mature human CNS [158].

The maturation state of iPSC‐derived neural cells represents another significant challenge for disease modeling applications. Most current differentiation protocols generate cells that resemble fetal or early postnatal stages of development rather than mature adult neurons and glia [159]. This developmental immaturity is particularly problematic for modeling age‐related neurodegenerative diseases, where pathological changes typically occur in mature neural networks over extended time periods [160]. The inability to achieve full cellular maturation in vitro may result in the absence of key disease phenotypes or the presence of developmental artifacts that do not reflect adult disease pathology [161].

The temporal limitations of iPSC models pose additional challenges for studying neurodegenerative diseases that develop over decades in human patients [162]. While some organoid systems can be maintained for extended periods, most current models exhibit pathological changes within weeks to months, which may not accurately reflect the slow progression of many rare neurological disorders [64]. This compressed timeline may lead to the identification of therapeutic targets or mechanisms that are relevant to acute cellular stress but not to the chronic degenerative processes that characterize human disease [163].

Technical reproducibility and standardization issues continue to plague iPSC‐based disease modeling efforts [164]. Variability in iPSC generation, maintenance, and differentiation protocols can lead to significant differences in experimental outcomes between laboratories and even between experiments within the same laboratory [165]. This variability is particularly problematic for rare disease research, where small effect sizes and limited patient samples make it essential to achieve high levels of experimental consistency and reproducibility [166].

The challenge of modeling complex genetic interactions and environmental factors represents another limitation of current iPSC approaches [167]. While iPSC models can capture the genetic background of individual patients, they cannot easily recapitulate the complex gene–environment interactions that contribute to disease development and progression in vivo [168]. Environmental factors such as aging, stress, infection, and lifestyle factors that may influence disease phenotype are difficult to model accurately in iPSC systems, potentially limiting their predictive value for therapeutic development [169].

Drug penetration and pharmacokinetic considerations present additional challenges for CNS drug discovery using iPSC models [170]. The blood‐brain barrier (BBB) represents a major obstacle for CNS drug development, with less than 2% of drugs successfully reaching the CNS [170]. Current iPSC models often lack functional BBB components, making it difficult to assess drug penetration and distribution properties that are critical for therapeutic efficacy in vivo [171]. This limitation may lead to the identification of compounds that show activity in iPSC models but fail to achieve therapeutic concentrations in the human brain [172].

The scalability limitations of complex 3D iPSC models pose challenges for high‐throughput drug screening applications [173]. While 2D iPSC models can be readily adapted for large‐scale screening efforts, the more biologically relevant 3D organoid systems are often labor‐intensive, expensive, and difficult to standardize for high‐throughput applications [174]. The interpretation and translation of findings from iPSC models to clinical applications remain challenging due to the lack of established correlations between in vitro phenotypes and clinical outcomes [175]. Future efforts to address these limitations will likely focus on developing more sophisticated culture systems that better recapitulate human brain complexity, improving cellular maturation protocols, and establishing standardized methodologies for reproducible results [176].

### 3.6. Use of CRISPR/Genome Editing in Disease Modeling

The integration of CRISPR/Cas9 genome editing with iPSC‐based disease modeling has transformed the study of RNDs by allowing precise genetic modification of human cells [177]. Isogenic control lines, introduced disease‐causing mutations, and corrected pathogenic variants can all be generated in a patient’s own genetic background, creating new opportunities to define disease mechanisms and test targeted interventions [178].

The system requires only two components: a Cas9 endonuclease and a single guide RNA whose 20‐nucleotide spacer base pairs with the target and positions the nuclease next to a protospacer‐adjacent motif [178]. Cas9 generates a double‐strand break that is resolved either by nonhomologous end joining, which introduces insertions or deletions and is used to create loss‐of‐function alleles, or by homology‐directed repair (HDR) from a supplied template, which permits precise correction or knock‐in [179]. HDR is inefficient relative to end joining, so iPSC protocols combine single‐stranded oligodeoxynucleotide donors with cell‐cycle synchronization to raise precise editing rates [180]. Transient nucleofection of preassembled Cas9–guide RNA ribonucleoprotein complexes is efficient and avoids the insertional risk of plasmid or lentiviral delivery [53]. Many RNDs are caused by single‐nucleotide changes. Base editors correct these directly, converting one base pair into another without cutting both DNA strands [181, 182]. Prime editing is more versatile, using a reverse transcriptase fused to a Cas9 nickase to introduce small insertions, small deletions, and any base substitution [183]. Neither approach depends on HDR, and neither produces the unwanted insertions and deletions seen with conventional editing.

The most important contribution of this technology is the isogenic control line [184]. Because matched lines differ only in the presence or absence of the disease‐causing variant, its effect can be separated from the rest of the patient’s genetic background [185]. This is especially valuable in rare neurological disorders, where few patient samples exist and genetic heterogeneity otherwise obscures genotype–phenotype relationships [186]. Editing has been applied to genes associated with Alzheimer’s disease, Parkinson’s disease, Huntington’s disease, and a range of rare genetic disorders [187, 188], and correction experiments in patient‐derived iPSCs have eliminated mutant protein production and rescued cellular phenotypes, establishing causality rather than association [189–191]. This is particularly important in RNDs, where animal models are often absent or unrepresentative [192, 193]. Table S2 summarizes genes modified by genome editing in iPSC models of the disorders discussed in this review, with the editing approach used and the phenotype interrogated.

CRISPR‐based functional genomics extends this beyond single genes to whole pathways [194]. Arrayed and pooled screening platforms have been applied to iPSC‐derived cell types [195, 196], and catalytically inactive Cas9 fused to repressor or activator domains (CRISPRi and CRISPRa) allows reversible, titratable control of gene dosage, now implemented as a screening platform in iPSC‐derived neurons [197, 198].

Validation of edited lines is not optional. Each candidate clone must be sequenced across the target locus to confirm the intended edit. Large on‐target deletions and loss of heterozygosity must also be excluded. Off‐target activity should be measured experimentally, using unbiased genome‐wide methods rather than prediction alone [199]. Genomic integrity should be reassessed after editing because human pluripotent stem cell cultures repeatedly acquire dominant‐negative TP53 and other growth‐advantageous mutations during expansion [200]. Several independently derived clones per genotype should be compared, so that clone‐specific artifacts are not mistaken for effects of the edited variant.

Limitations with CRISPR genome editing still remain. Off‐target editing is a persistent concern, particularly when several modifications are made simultaneously or when working at complex loci [201–203]. In vivo application is further constrained by the difficulty of delivering editing components to defined cell types in the brain, which limits direct therapeutic translation for most neurological disorders [204]. Improved guide RNA design, higher‐fidelity Cas variants, and new editing modalities are expected to address several of these constraints [205].

## 4. Current Use of iPSCs for Modeling of RNDs

iPSCs, reprogrammed from somatic cells to the pluripotent state by introducing key transcription factors, can differentiate into virtually any cell type, including neurons, glia, and other brain cell types. The ability to derive iPSCs from patients allows for disease modeling that is both patient‐specific and genetically accurate. One of the most significant contributions of the iPSC technology is its capacity to generate patient‐specific cell models [206]. For rare genetic neurological diseases, where mutations may vary from patient to patient, iPSCs allow researchers to preserve the unique genetic background of each individual.

Pediatric neurodevelopmental disorders represent some of the most challenging conditions to study in traditional research frameworks. That makes the RNDs ideal candidates for iPSC‐based studies as it is quite difficult to collect human neural tissue and also model the complexity of the mammalian nervous system.

There are several breakthrough studies that have demonstrated the power of this approach. Down syndrome has been successfully modeled by using iPSCs derived from patients, which revealed the complex and genome‐wide changes in neural cells associated with prolonged cell cycle, reduced cell growth, and perturbed energy metabolism [207–209]. In this context, patient‐specific iPSC models can be valuable in improving our understanding of mechanisms related to phenotypic variability, neurodevelopment, progression, and severity of this condition.

Another rare disorder, Rett syndrome, has been extensively studied using patient‐derived iPSCs. This rare X‐linked neurodevelopmental disorder primarily affects females and involves normal early development, followed by regression with loss of acquired motor and communication skills. Nearly all cases result from mutations in the *MECP2* gene, leading to reduced or dysfunctional MeCP2, a protein essential for brain development and gene regulation. Patient‐derived iPSCs have been used to model *MECP2* mutations, impacting synaptic function and neuronal phenotype, offering insight into disease mechanisms, thus providing a powerful system for studying Rett syndrome by enabling investigation of human‐specific biology, genetic manipulation, and disease mechanisms that cannot be easily modeled in vivo [90–98]. While challenges such as variability between patient‐derived lines and culture conditions remain, iPSCs are invaluable for exploring the cellular and molecular basis of Rett syndrome and for advancing the development of new therapeutic strategies.

SMA, caused by mutations in the SMN1 gene, represents one of the most successful examples of iPSC disease modeling [70, 71]. In these studies, iPSC‐derived motor neurons from SMA patients showed selective reduction in number and size compared to control cells, suggesting that SMA motor neurons developed normally but were more susceptible to degeneration. Importantly, treatment with valproic acid and tobramycin led to increased SMN protein levels, providing proof of principle for drug screening applications. In a recent study by Giadone et al. [72], iPSCs were utilized to create a high‐throughput screening platform and identified numerous compounds that modulate disease‐related gene expression.

Another motor neuron disorder, hereditary spastic paraplegia, caused by autosomal dominant mutations in the *SPAST* gene, was modeled by using the iPSCs from patients with SPG3a or SPG4 types of mutations. iPSCs were differentiated into motor neurons and neural cells and exhibited axonal swellings, impaired neuromuscular junctions, and microtubule‐associated defects. This enabled the investigation into the mechanisms of axonal degeneration and for comparative studies and drug screening [73]. In another study, it was demonstrated that inhibition of microRNA *miR-33a* ameliorated the pathological phenotype in hereditary spastic paraplegia patient–based iPSC–derived cortical neurons [74]. Miller–Dieker syndrome is a rare genetic disorder (incidence 1 in 50,000–100,000 patients) causing fatal brain malformations. It has been modeled using brain organoids derived from patient iPSCs in a study that revealed that many neural stem cells die off at early stages of development, and others exhibit defects in cell movement and cell division, providing insights into how the genetics of this syndrome lead to lissencephaly [131].

Friedreich’s ataxia is a rare neurodegenerative disorder, affecting ~1 in 50,000 people worldwide, with autosomal recessive inheritance and caused by GAA repeat expansions in the *FXN* gene. Dorsal root ganglia organoids, neurons, and other appropriate cell types, vulnerable to *FXN* deficiency, were differentiated from patient‐derived iPSCs and used for disease mechanism investigation and testing of drug candidates [75–78]. iPSC models helped identify protein kinases (ROCK1 and ROCK2) as therapeutic targets, thus proposing that inhibiting these kinases with FDA‐approved drugs could reactivate the silenced disease–causing gene [79].

Among rare neuromuscular junction and muscle disorders, Pompe disease is caused by mutations in the *GAA* gene with autosomal recessive inheritance, with an overall global birth incidence of 1 in 40,000 live births. Somatic cells from Pompe patients were reprogrammed to iPSCs and then differentiated into skeletal myocytes, cardiomyocytes, hepatocytes, and neurons. Studies using these models showed disease‐typical glycogen accumulation and enzyme deficiency and were used for phenotypic assays, evaluating new therapeutics, and insights into metabolic dysfunction [80–82].

Dravet syndrome, a rare neurodevelopmental disorder (incidence estimated at around 1 in every 15,700 live births), is an epileptic syndrome with high risk of sudden unexpected death. It is most often caused by, typically, de novo mutations in the *SCN1A* gene, which encodes Nav1.1 protein (subunit of voltage‐sensitive sodium channel). Patient‐derived iPSCs were used to study neuronal excitability and cardiac arrhythmia risk, providing direct insight into multisystem involvement in this syndrome [99–103, 130]. These cell lines were used to establish ventral forebrain organoids that could provide insights into the pathology behind Nav1.1 haploinsufficiency and offer platform for screening of drugs with potential to remedy the deficits associated with the disease.

CMT, the most common inherited peripheral nervous system disorder (it affects ~1 in 2,500 people worldwide), is caused by mutations in several genes (e.g., *PMP22*, *MPZ*, and *GJB1*) and in 40%–50% of cases caused by the duplication of the *PMP22* gene on chromosome 17 (type 1A). Patient‐specific iPSC lines were derived and differentiated into neural crest and Schwann cell precursors, which brought about systems able to recreate CMT1A pathophysiology and allowed study of myelination defects and screening for mutation‐correcting strategies [85–88, 104–112]. iPSCs were also generated from skin fibroblasts, lymphoblastoid cells, and T cells of patients with various CMT and differentiated into neurons and glial cells. These models reproduced subtype‐specific pathological phenotypes, including mitochondrial, bioenergetic, and neurofilament abnormalities, as well as Schwann cell defects, with some studies in which iPSCs were used to obtain neurospheres and spinal spheroids. Genome editing (CRISPR/Cas9) corrected mutations and rescued disease phenotypes, while pharmacological interventions partially improved the dysfunction.

Sturge–Weber syndrome and tuberous sclerosis complex (TSC) are neurocutaneous disorders, that is, genetic conditions characterized by abnormalities in both the nervous system and the skin. While iPSC‐based 3D co‐culture models for Sturge–Weber syndrome have been suggested as highly relevant for disease modeling, there are currently no examples of patient‐derived iPSC models related to this condition. Contrarily, there are examples of patient iPSC genome‐edited at TSC1/TSC2 loci that have been differentiated into neural progenitor and neural crest cells (NCCs) [89, 113–126]. Patient‐specific iPSCs captured key TSC hallmarks: mTORC1 dysregulation, altered neuronal network activity, and tuber‐like transcriptional changes. Thus, they represent genetically accurate models to study cellular and molecular pathology and to perform drug screenings relevant to the neurological symptoms of TSC.

As for other RNDs, such as ataxia‐telangiectasia, Leigh syndrome, Wilson’s disease, and genetic dystonia, while iPSC models for these conditions are mentioned in broader iPSC literature as an area of active research, specific detailed protocol examples are less commonly published compared to the disorders above. A summary on the use of iPSCs for the modeling of selected RNDs can be found in Table 1.

## 5. Future Opportunities for the Application of iPSCs in Rare Neurological Condition Research

Since the first generation of human iPSCs, a vast number of patient‐specific iPSC lines have been developed. In recent years, research involving iPSCs has expanded rapidly, with their potential applications in disease modeling, drug screening, and cell‐based therapies gaining widespread recognition. Compared to human embryonic stem cells, iPSCs offer distinct advantages, including the absence of ethical concerns and a reduced risk of immune rejection. While iPSCs are being explored for direct therapeutic use in cell replacement strategies, disease modeling remains one of the most valuable application of this technology [210].

In the future, it is expected that repositories of pluripotent stem cells will be established, enabling the modeling of various monogenic, chromosomal, and complex genetic diseases. The first step involves generating cell lines by introducing or correcting relevant mutations (in some cases, introducing different mutations within the same gene) to create isogenic cell lines. By using patient‐derived iPSCs, it is possible to introduce mutations in multiple genes that are involved in the patient’s disease [14]. The year 2026 will mark two decades since the discovery of iPSCs. Over the past 20 years, the iPSC technology has made remarkable advances, yet ongoing efforts continue to expand and refine its full potential. Although significant progress has been made in differentiating iPSCs into various specialized cell types, these protocols remain under continuous development. Considerable efforts have already been made in the creation of cell‐based therapies for neurological and cardiac disorders, and future therapeutic applications are expected to encompass a broader spectrum of diseases. Several ongoing clinical trials are anticipated to yield approved therapies in the near future. Upon regulatory approval, such treatments will make regenerative medicine more accessible, offering more effective and more personalized therapy for patients. The continued collaboration between basic and translational research is expected to accelerate the advancement of cell therapy. One of the most compelling advantages of the iPSC technology lies in its potential for autologous transplantation. Using a patient’s own corrected cells for treatment substantially minimizes the risk of immune rejection. There is growing optimism that therapies based on the principle of “curing one’s own disease with one’s own cells” will become a standard approach in personalized medicine. That kind of approach could transform the field of regenerative medicine, offering significant improvements in treatment outcomes and enhancing the quality of life for many patients [211]. The biological responses observed in preclinical animal models may not fully predict outcomes in humans, and our understanding of iPSC differentiation processes is still incomplete. To advance toward safe and effective clinical applications, future research should prioritize the refinement of differentiation protocols for iPSC‐derived neural cells, with a focus on ensuring their long‐term survival, functional integration within host tissues, and therapeutic efficacy in vivo [212].

## 6. Conclusions

Human iPSCs represent a transformative advancement in the study of RNDs, offering unprecedented fidelity in modeling human‐specific pathophysiology. Their capacity to recapitulate patient‐specific genotypes, coupled with CRISPR‐based gene editing and advanced 3D culture systems, positions iPSC models at the frontier of translational neuroscience. However, significant challenges, ranging from incomplete cellular maturation to limitations in modeling long‐term disease trajectories, must be addressed to ensure clinical relevance. The field is now poised at a critical juncture; with rigorous standardization, cross‐disciplinary collaboration, and ethical commitment, iPSC technologies can redefine the landscape of rare disease research. Future integration into drug discovery and regenerative medicine pipelines holds promise not only for accelerating therapeutic innovation but also for personalizing treatment strategies for previously untreatable conditions. Realizing this vision demands sustained investment and a cohesive global effort.

**Nomenclature**
CNS: Central nervous system
iPSCs: Induced pluripotent stem cells
EMA COMP: Committee for Orphan Medicinal Products
RNDs: Rare neurological diseases
SMA: Spinal muscular atrophy
CMT: Charcot‐Marie‐Tooth disease
TSC: Tuberous sclerosis complex
FDA: Food and Drug Administration
2D: Two‐dimensional
3D: Three‐dimensional

## Author Contributions

Conceptualization: Kristina Pilipović and Anja Harej Hrkać. Writing – original draft preparation: Anja Harej Hrkać, Mukesh Varshney, Valentino Rački, Andrej Belančić, Jasna Kriz, Dinko Vitezić, Jasenka Mršić‐Pelčić, Kristina Pilipović, and Ganesh Acharya. Writing – review and editing: Kristina Pilipović, Anja Harej Hrkać, Mukesh Varshney, and Jasenka Mršić‐Pelčić. Visualization: Mukesh Varshney. Supervision: Jasna Kriz, Dinko Vitezić, and Jasenka Mršić‐Pelčić.

## Funding

This research was funded by the University of Rijeka (Grant uniri‐mzi‐25‐6 to Kristina Pilipović and Grant uniri‐mladi‐biomed‐23‐35 to Anja Harej Hrkać). Mukesh Varshney and Ganesh Acharya acknowledge the support by Sweden’s Innovation Agency, VINNOVA (Grant 202100–2973), and Karolinska Institutet research grants.

## Disclosure

All authors have read and agreed to the published version of the manuscript.

## Conflicts of Interest

The authors declare no conflicts of interest.

## Supporting Information

Additional supporting information can be found online in the Supporting Information section.

## Supporting information


**Supporting Information** Table S1: Representative protocols for generating 2D neural cells and 3D organoids from human iPSCs, including cell/model types, core differentiation strategies, approximate durations, and major applications. Table S2: Genes modified by genome editing in iPSC models of rare neurological disorders, detailing the associated disorder, editing approach, model/cell type, and purpose of the genetic modification.

## Data Availability

Data sharing is not applicable to this article as no datasets were generated or analyzed during the current study.
