# Supplementary material for: Human Induced Pluripotent Stem Cells in Rare Neurological Disorders: Opportunities and Challenges for Disease Modeling and Drug Discovery
Source: Stem Cells Int. 2026 Oct 21;2026:1130043. doi: 10.1155/sci/1130043 (PMC13685662; doi:10.1155/sci/1130043)
Supplement: Supplementary file 1 [file SCI-2026-1130043-s001.docx]

**Supplementary Table 1.** Representative protocols for generating 2D neural cells and 3D organoids from human iPSCs. Durations are approximate and protocol-dependent.

| Format | Cell type / model | Core strategy | Approx. duration | Main application | Ref. |
| --- | --- | --- | --- | --- | --- |
| 2D | Cortical glutamatergic neurons | Dual-SMAD inhibition → dorsal forebrain progenitors | 1–3 months | Cell-autonomous phenotypes, drug screening | 80, 195 |
| 2D | Forebrain GABAergic interneurons | Dual-SMAD + SHH agonist, WNT inhibition (MGE-like) | 2–4 months | Excitation–inhibition phenotypes, epilepsy models | 196 |
| 2D | Spinal motor neurons | Dual-SMAD + RA + SHH agonist | 3–5 weeks | SMA, hereditary spastic paraplegia, CMT | 197 |
| 2D | Midbrain dopaminergic neurons | Floor-plate induction (SHH, FGF8, WNT activation) | 1–2 months | Dopaminergic pharmacology and toxicology | 198, 56 |
| 2D | Induced excitatory neurons | Inducible NGN2 expression | 1–2 weeks | Scalable screening, isogenic panels | 199, 200 |
| 2D | Astrocytes | Expansion of neural progenitors + gliogenic maturation | 2–6 months | Neuron–glia co-culture, non-cell-autonomous effects | 201 |
| 2D | Microglia | Haematopoietic progenitor intermediate + M-CSF/IL-34 | 5–8 weeks | Neuroinflammation | 202 |
| 3D | Unguided cerebral organoids | Embryoid body → neuroectoderm → matrix embedding → agitation | 1 month to >6 months | Neurodevelopmental architecture, progenitor dynamics | 61, 40 |
| 3D | Region-specific (guided) organoids | Defined morphogen patterning in suspension | 1 month to >6 months | Region-restricted phenotypes, improved reproducibility | 98, 203 |
| 3D | Assembloids | Fusion of two region-specific organoids | +1 month after fusion | Interneuron migration, inter-regional connectivity | 203 |
| 3D | ALI organoid slices | Sectioning and air–liquid interface culture | >2 months | Axon tract outgrowth, functional output | 204 |
| 3D | Vascularised organoids | Endothelial co-culture or induced ETV2 expression | 1–3 months | Nutrient delivery, barrier-related questions | 205 |
| 3D | Organoid co-culture | Seeding exogenous cells onto organoids | Variable | Tumour invasion, cell-based therapy testing | 63 |

*Abbreviations:* SHH, sonic hedgehog; RA, retinoic acid; MGE, medial ganglionic eminence; TF, transcription factor; ALI, air–liquid interface.

**Supplementary Table 2.** Genes modified by genome editing in iPSC models of rare neurological disorders discussed in this review.

| Gene | Disorder | Editing approach | Model/ derived cell type | Purpose | Ref |
| --- | --- | --- | --- | --- | --- |
| *SMN2* → *SMN1* | SMA | CRISPR/Cas12a (Cpf1) with ssODN, seamless conversion | Patient iPSCs | Restore full-length SMN expression | 214 |
| *SPAST* | Hereditary spastic paraplegia (SPG4) | CRISPR/Cas9 knockout | iPSC lines | Generate loss-of-function model | 215 |
| *FXN* | Friedreich's ataxia | Targeted frataxin gene editing | DRG organoid-derived sensory neurons | Rescue disease pathology | 152 |
| *SCN1A* | Dravet syndrome/ epilepsy | Isogenic pair generation by editing a patient variant | iPSC-derived neurons | Attribute functional deficits to the variant | 216 |
| *NEFL* | CMT type 2E | Allele-specific CRISPR/Cas9 editing | Patient iPSC-derived neurons | Rescue pathology | 165 |
| *GARS1* | CMT type 2D | CRISPR/Cas9-mediated gene repair | Patient iPSC line | Correct the G294R variant | 168 |
| *PMP22* | CMT type 1A | AAV-delivered genome editing | Patient iPSC-derived Schwann cells | Reduce gene dosage, rescue features | 176 |
| *MECP2* | Rett syndrome | Precise CRISPR/Cas9 modification | Human iPSCs | Generate mutant and corrected lines | 145 |
| *TSC2* | Tuberous sclerosis complex | Isogenic line generation | Patient-derived neural progenitors | Isolate variant effect on neurodevelopment | 185 |
| *SOD1* | ALS | Targeted genetic correction | Patient iPSC-derived motor neurons | Identify variant-dependent pathways | 121 |
| *HTT* | Huntington's disease | Homologous recombination-mediated correction | Patient iPSCs | Reverse disease-associated phenotypes | 122 |

*Abbreviations:* SMA, spinal muscular atrophy; CMT, Charcot–Marie–Tooth disease; ALS, amyotrophic lateral sclerosis; NHEJ, non-homologous end joining; HDR, homology-directed repair; ssODN, single-stranded oligodeoxynucleotide; DRG, dorsal root ganglion.
